# Lipin3 deficiency promotes hepatocyte ferroptosis and pyroptosis via activating JAK1-STAT3 pathway during acetaminophen induced acute liver injury

**DOI:** 10.1186/s43556-025-00317-z

**Published:** 2025-10-10

**Authors:** Yu-Xing Liu, Qian Wang, Zi-Yu Xiangyang, Jie-Yi Long, Hao Huang, Liang-Liang Fan

**Affiliations:** 1 Department of Cell Biology, School of Life Science, Central South University, Changsha, 410013 China; 2 Department of Nephrology, Xiangya Hospital of Central South University, Changsha, 410028 China

**Keywords:** Acute liver injury, Lipin3, Ferroptosis, Pyroptosis

## Abstract

Lipin3 belongs to the Lipin protein family and is pivotal in modulating lipid homeostasis, inflammatory signaling, and lineage commitment. However, research on Lipin3 is limited, and its role in liver diseases remains poorly defined. This study investigated the function of Lipin3 in acetaminophen (APAP)-induced acute liver injury (ALI). Lipin3 expression was analyzed in public ALI datasets, ALI patients, APAP-challenged mouse models and primary hepatocytes. *Lpin3-*knockout (*Lpin3-*KO) mice and adeno-associated virus (AAV)-overexpressing *Lpin3* mice were generated to assess the pathophysiological role of Lipin3. Mechanistic studies, including mass spectrometry, coimmunoprecipitation, and bioinformatics prediction, were conducted in primary hepatocytes and HepG2 cells. Our key findings were as follows: Lipin3 levels were markedly reduced in ALI patients, APAP-treated mice, and hepatocytes. Compared with wild-type mice, *Lpin3-*KO mice exhibited exacerbated ALI severity after post-APAP exposure. Lipin3 deficiency promoted hepatocyte ferroptosis (via lipid peroxidation/ACSL4) and pyroptosis (via GSDME cleavage). Mechanistically, Lipin3 directly interacts with JAK1 to suppress its phosphorylation, thereby inhibiting STAT3-driven activation of ACSL4 (ferroptosis) and GSDME (pyroptosis). Lipin3 overexpression mitigated APAP-induced hepatocyte ferroptosis and pyroptosis, thereby alleviating ALI. Our results demonstrate that Lipin3 depletion aggravates ALI through the dual regulation of ferroptosis and pyroptosis through the JAK1-STAT3 axis, suggesting that Lipin3 is a potential therapeutic target for APAP-induced liver injury.

**Supplementary Information:**

The online version contains supplementary material available at 10.1186/s43556-025-00317-z.

## Introduction

Acute liver injury (ALI) refers to the sudden and severe impairment of liver function that can occur because of various causes, including drug overdose, infections, autoimmune reactions, or other liver diseases. As a globally important public health issue, ALI mainly results from acetaminophen (APAP) abuse in developed countries, and APAP-induced ALI has become one of the crucial causes of liver-related death worldwide [1]. The pathogenesis of ALI is characterized by extensive cell death in the liver, thereby triggering a cascade of immune responses [2]. Apoptosis, necroptosis, and autophagy are well-documented cell death pathways in APAP-induced ALI [3]. However, ferroptosis and pyroptosis have rarely been reported in this context.

Lőrincz et al. first described in 2015 that ferroptosis was involved in APAP-induced cell death in hepatocytes [4]. The depletion of glutathione, iron accumulation, and lipid peroxidation have long been recognized as the fundamental mechanisms underlying APAP-induced ALI [5, 6]. Multiple studies have suggested that ferrostatin 1, a specific ferroptosis inhibitor, can protect hepatocytes from APAP and alleviate APAP-induced ALI in a dose-dependent manner [7]. Recent studies have shown that lipid peroxidation is a critical driver of ferroptosis, particularly under conditions of iron overload or severe impairment of antioxidant defenses. When iron overload occurs in the context of APAP exposure, excess iron can catalyze the formation of nitrotyrosine and promote lipid peroxidation, both of which contribute to cell death [8].

In 2019, Yang et al. reported that liver injury was exacerbated in gasdermin D-deficient mice following APAP administration [9]. In 2021, Wang et al. first reported that pyroptosis occurred in APAP-induced AlI and suggested that peroxiredoxin 3 deficiency may contribute by producing excessive amounts of mitochondrial reactive oxygen species, which further trigger pyroptosis by activating the NOD-like receptor protein 3 (NLRP3) inflammasome, and that knockdown of NLRP3 expression reduced the levels of all pyroptosis parameters and overall liver injury [10]. Multiple compounds or molecules were subsequently found to be involved in hepatocyte or macrophage pyroptosis through the regulation of the NLRP3-Caspase 1 pathway [11, 12]. Recently, two studies have indicated that Caspase 11-dependent noncanonical pyroptosis and gasdermin E (GSDME)-dependent noncanonical pyroptosis in hepatocytes play crucial roles in APAP induced ALI [13, 14].

Lipin3 belongs to the Lipin protein family, whose members are important for regulating lipid homeostasis, inflammatory signaling, and lineage commitment [15]. Lipin family members share a bipartite architecture: an N-terminal module (N-LIP) and a C-terminal module (C-LIP). The N-LIP segment mediates enzymatic catalysis, nuclear import, and association with protein phosphatase-1cγ, while the C-LIP segment modulates phosphatidate phosphatase function and acts as a transcriptional coactivator [16]. Previous research has shown that Lipin3, in conjunction with Lipin1, influences adiposity in vivo. Additionally, Lipin3 collaborates with Lipin2 to maintain phospholipid homeostasis and regulate chylomicron synthesis [17–19]. Our group was the first to identify Lipin3 as a novel pathogenic gene responsible for obesity and hypertriglyceridemia [19]. However, the role of Lipin3 in liver diseases remains unclear.

Here, we aimed to explore the relationship between Lipin 3 and APAP-induced ALI, especially the underlying molecular mechanisms involved. *Lpin3* gene knockout and adeno-associated virus (AAV)-overexpressing *Lpin3* mouse models were constructed for APAP intervention to confirm that Lipin3 can regulate APAP-induced ALI. Primary cultured hepatocytes and HepG2 cells were used to confirm that Lipin3 could regulate ferroptosis and pyroptosis in hepatocytes after APAP induction. The potential molecular mechanisms were explained through mass spectrometry, co-IP, bioinformatics prediction and STAT3 inhibitor treatment of cells. Finally, our study suggested that Lipin3 deficiency can induce pyroptosis mediated by GSDME and ferroptosis mediated by ACSL4 via the JAK1-STAT3 signaling pathway in hepatocytes, thereby exacerbating ALI induced by APAP. Our research suggested that a reduction in Lipin3 might predispose individuals to APAP-induced ALI, highlighting Lipin3 as a potential therapeutic target.

## Results

### Low expression of Lipin3 is associated with ALI

**Fig. 1:**
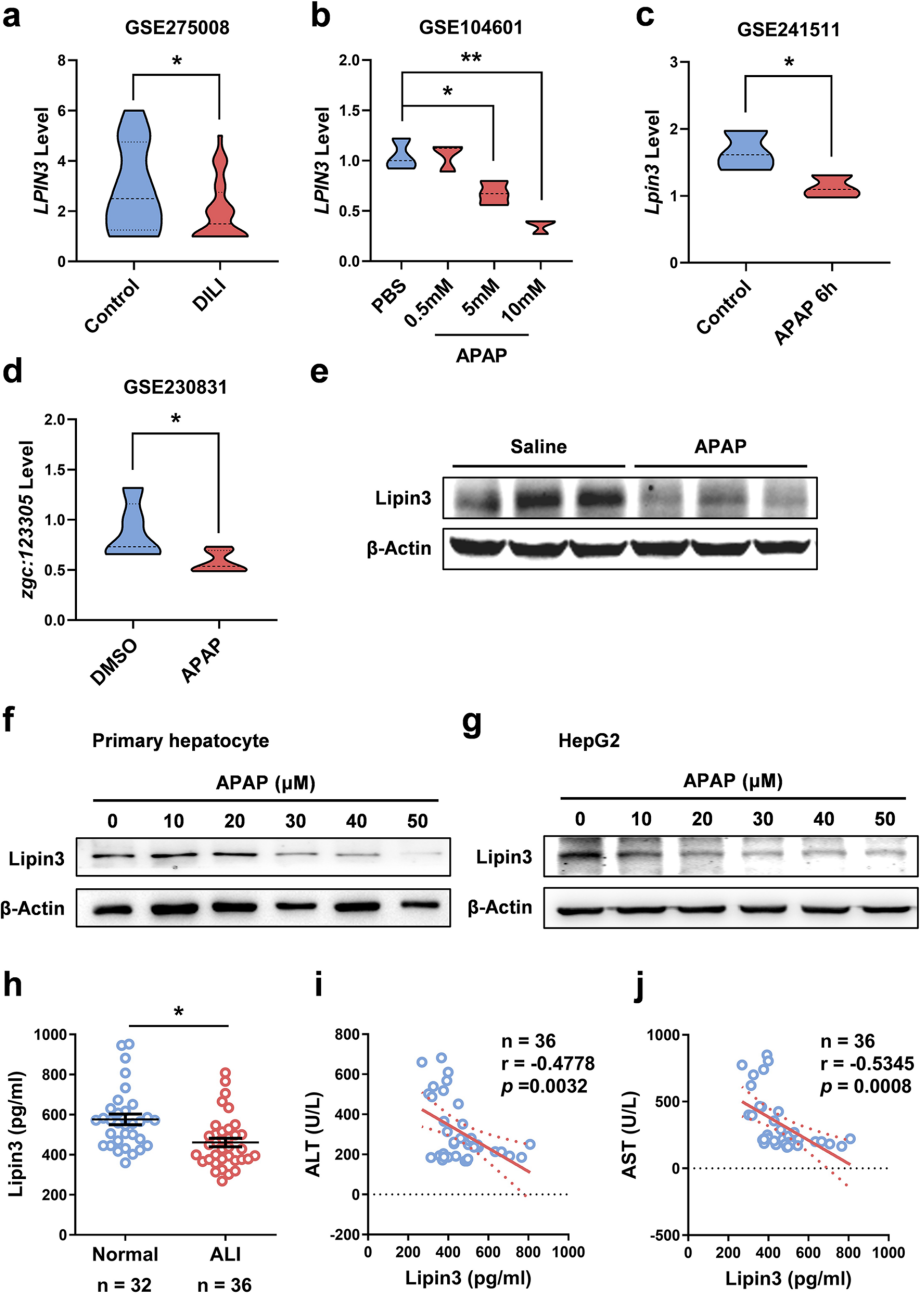
Link between low expression of Lipin3 and APAP-induced ALI. (**a**) The *LPIN3* gene expression in DILI patients (GSE275008). (**b**) The *LPIN3* gene expression in APAP-treated 3-D human liver microtissues (GSE104601). (**c**) The *Lpin3* gene expression in mouse livers (GSE241511) after APAP treatment. (**d**) The *zgc:123,305* gene expression in zebrafish (GSE230831) exposure in APAP. (**e**) WB analysis showing the expression levels of Lipin3 in mice treated with or without APAP. WB analysis showing the expression levels of Lipin3 in primary hepatocytes (**f**) and HepG2 cells (**g**) treated with different concentrations of APAP. (**h**) The expression of Lipin3 in the peripheral serum of healthy individuals and ALI patients. Scatter plots showing the correlation between serum Lipin3 levels and serum ALT levels (**i**) and serum AST levels (**j**). Correlation coefficient r and *p* value were calculated by the Spearman’s rank correlation coefficient test. *p < 0.05, **p < 0.01

To explore the potential role of Lipin3 in ALI, we initially analyzed its expression profiles in public database. We analyzed the GSE275008 blood transcriptome dataset from patients with drug-induced liver injury (DILI) and found that *LPIN3* mRNA expression was markedly lower in these patients than in matched controls (Fig. 1a). In the GSE104601 dataset, APAP-treated 3-D human liver microtissues also exhibited a significant reduction in *LPIN3* mRNA expression (Fig. 1b). Similarly, the GSE241511 liver dataset from APAP-injured mice revealed a pronounced decrease in *Lpin3* expression compared with that in saline-treated controls (Fig. 1c). Similarly, APAP exposure in the zebrafish dataset GSE230831 markedly downregulated *zgc:123,305*, the zebrafish ortholog of human *LPIN3* (Fig. 1d). We then generated an ALI mouse model via intraperitoneal injection of APAP (Figure S1a-c), and WB analysis revealed a reduction in Lipin3 expression in APAP-treated mouse liver tissue (Fig. 1e). We treated primary hepatocytes and HepG2 cells in vitro with APAP. WB analysis revealed that Lipin3 expression decreased as the APAP dose increased (Fig. 1f and g). In addition, we enrolled serum samples from 32 Healthy controls and 36 patients with ALI (Table S1). ELISA revealed that serum Lipin3 levels were significantly lower in patients with ALI than in healthy controls, and this reduction was evident even in those whose ALI was specifically triggered by APAP (Fig. 1h and Figure S2). Moreover, serum Lipin3 levels progressively decreased with worsening hepatic injury and strongly inversely correlated with alanine aminotransferase (ALT) and aspartate aminotransferase (AST) levels (Fig. 1i and j). Data from public databases, murine models, cultured cells, and patient samples uniformly indicated a significant association between Lipin3 down-regulation and ALI pathogenesis.

### Lipin3 deficiency exacerbates APAP induced ALI

**Fig. 2:**
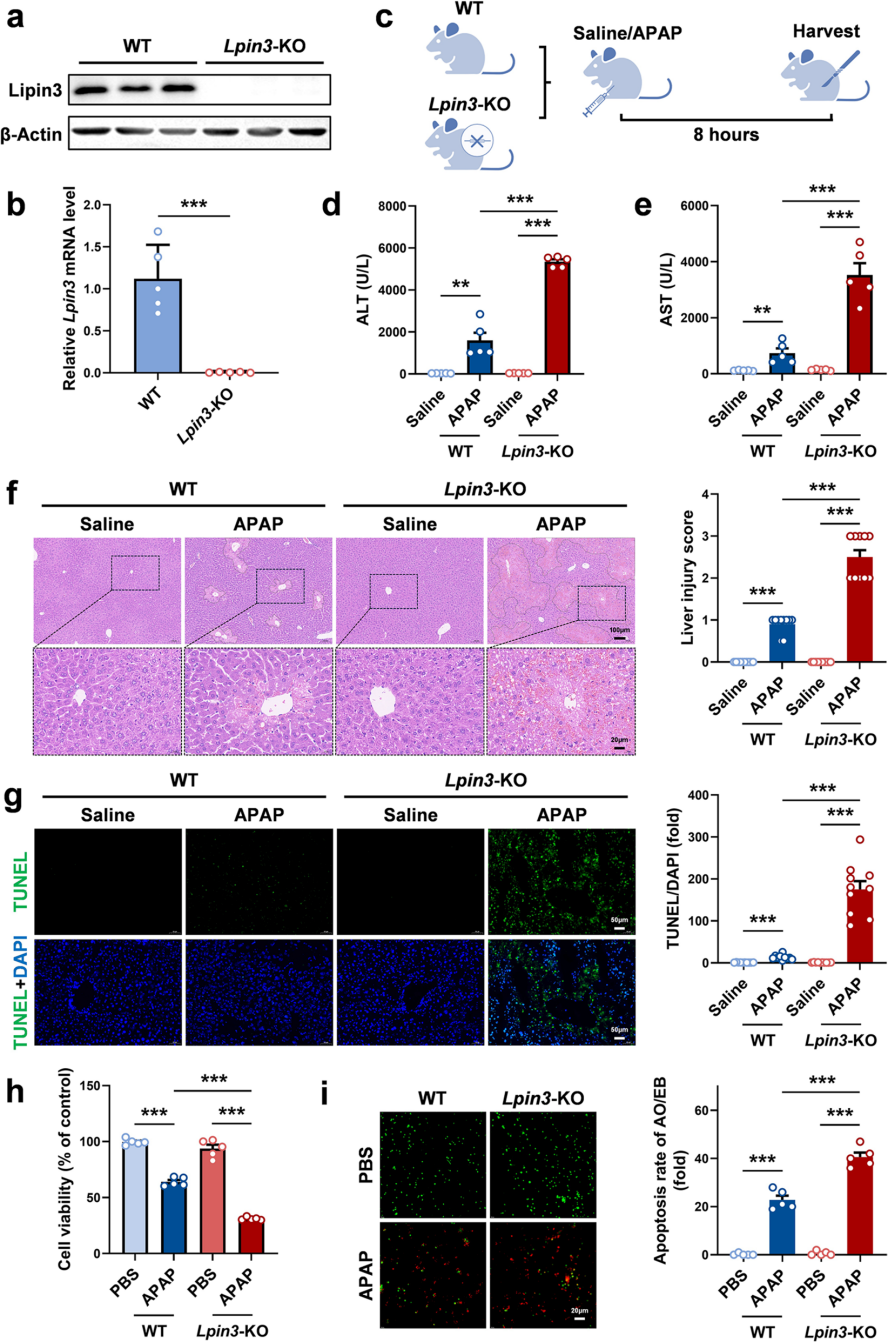
Lipin3 deficiency exacerbates APAP-induced AKI. WB analysis (**a**) and qPCR (**b**) verified the successful generation of *Lpin3*-KO mice. (**c**) Flow chart of animal study design. Peripheral blood ALT (**d**) and AST (**e**) levels of WT mice (*n* = 5) and *Lpin3*-KO mice (*n* = 5) treated with or without APAP. (**f**) HE staining analysis showing the liver injury in WT mice (*n* = 5) and *Lpin3*-KO mice (*n* = 5) treated with or without APAP. (**g**) The TUNEL staining analysis showing the apoptosis in the liver of WT mice (*n* = 5) and *Lpin3*-KO mice (*n* = 5) treated with or without APAP. The fluorescence intensity in the graphs was calculated using Image J software analysis. (**h**) CCK8 analysis showing the cell viability of WT and *Lpin3*-KO primary hepatocytes treated with or without APAP. (**i**) AO/EB staining and statistical analysis of apoptosis rates in each primary hepatocytes group. **p < 0.01, ***p < 0.001

To elucidate Lipin3’s role in ALI, we generated *Lpin3-*KO mice (Fig. 2a and b). Ten-week-old male mice were subjected to ALI induction via intraperitoneal injection of APAP (Fig. 2c). Mouse serum ELISA revealed that APAP elevated ALT and AST levels, and *Lpin3*-KO mice exhibited markedly higher surges than WT controls (Fig. 2d and e). HE staining revealed no obvious differences in liver tissue between the WT and *Lpin3-*KO groups treated with saline. After APAP challenge, the livers of the *Lpin3*-KO group exhibited significantly more extensive necrosis than those of the WT group (Fig. 2f). Moreover, WB analysis revealed that the levels of HMGB1, an important biomarker of ALI, increased more overtly in the liver tissues of the *Lpin3-*KO group than in those of the WT group after APAP treatment (Figure S3). TUNEL staining of liver tissues also revealed that APAP provoked markedly more hepatocyte apoptosis in the *Lpin3-*KO group than in the WT group (Fig. 2g). Finally, we isolated primary hepatocytes as described previously. The results of the CCK8 assay demonstrated that *Lpin3-*KO cells displayed lower cell viability than WT cells did in an APAP concentration-dependent manner (Figure S4a and b; Fig. 2h). AO/EB staining also revealed that compared with WT cells, *Lpin3-*KO cells clearly increased the rate of cell death after APAP treatment (Fig. 2i). These findings indicated that Lipin3 deficiency promoted cell death of hepatocytes and exacerbated ALI after APAP treatment.

### Lipin3 deficiency activates ferroptosis in APAP-induced ALI

**Fig. 3:**
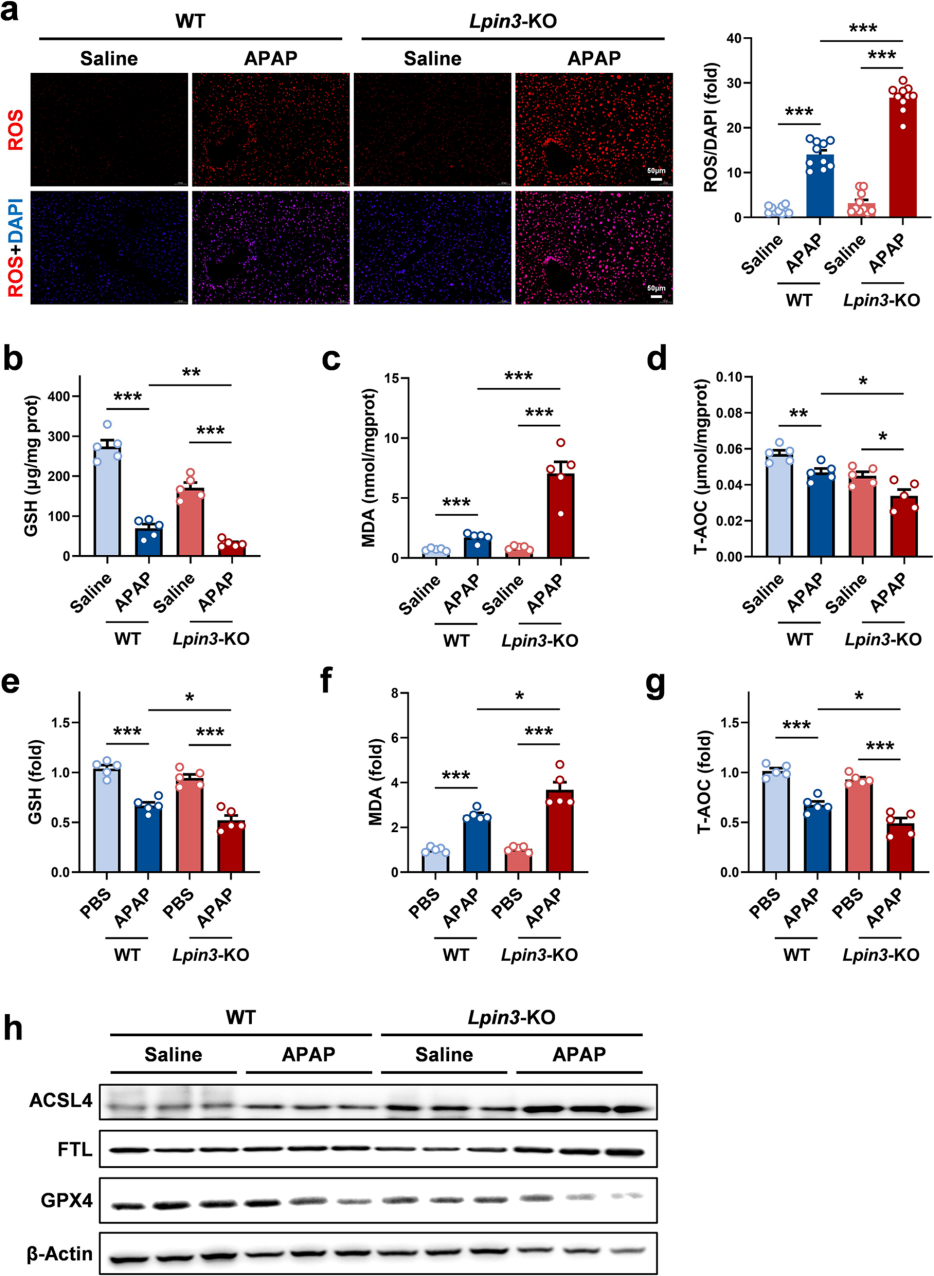
Lipin3 deficiency may activate the ferroptosis in APAP induced ALI. (**a**) The DCF staining analysis showing the ROS levels in the liver of WT mice (*n* = 5) and *Lpin3*-KO mice (*n* = 5) treated with or without APAP. (**b**) GSH levels, (**c**) MDA levels and (**d**) T-AOC levels in the liver of WT mice (*n* = 5) and *Lpin3-*KO mice (*n* = 5) treated with or without APAP. (**e**) GSH levels, (**f**) MDA levels and (**g**) T-AOC levels in WT and *Lpin3*-KO primary hepatocytes treated with or without APAP. (**h**) WB analysis revealed the expressions of ACSL4, FTL and GPX4 in the liver of WT mice (*n* = 5) and *Lpin3*-KO mice (*n* = 5) treated with or without APAP. *p < 0.05, **p < 0.01, ***p < 0.001

In the liver, the accumulation of N-acetyl-4-benzoquinone imine (NAPQI), a metabolite of APAP, leads to the depletion of glutathione (GSH), inducing ferroptosis in hepatocytes, and subsequently causing ALI [6]. Therefore, we assessed the extent of ferroptosis in hepatic samples from every experimental group. Immunofluorescence staining of liver tissues revealed that compared with those in the WT group, there was a significant accumulation of reactive oxygen species (ROS) in the liver tissues of *Lpin3-*KO mice after APAP treatment (Fig. 3a). Biochemical assays revealed that APAP treatment decreased the GSH level and increased the malondialdehyde (MDA) level in mouse liver tissue. Compared with WT mice, *Lpin3-*KO mice exhibited further decreases in GSH levels and increases in MDA levels in liver tissues after APAP treatment (Fig. 3b and c). Additionally, the total antioxidant capacity (T-AOC) was lower in *Lpin3-*KO mice than in WT mice (Fig. 3d). Consistent results were obtained in vitro when primary hepatocytes were exposed to APAP (Fig. 3e-g). Moreover, WB analysis demonstrated that, following APAP administration, the expression of two ferroptosis markers ACSL4 and GPX4, differed sharply between genotypes: the expression of ACSL4 was markedly greater and the expression of GPX4 was markedly lower in *Lpin3*-KO livers than in those of their WT counterparts (Fig. 3h). These results suggest that severe ferroptosis occurred in the liver of *Lpin3-*KO mice treated with APAP.

**Fig. 4:**
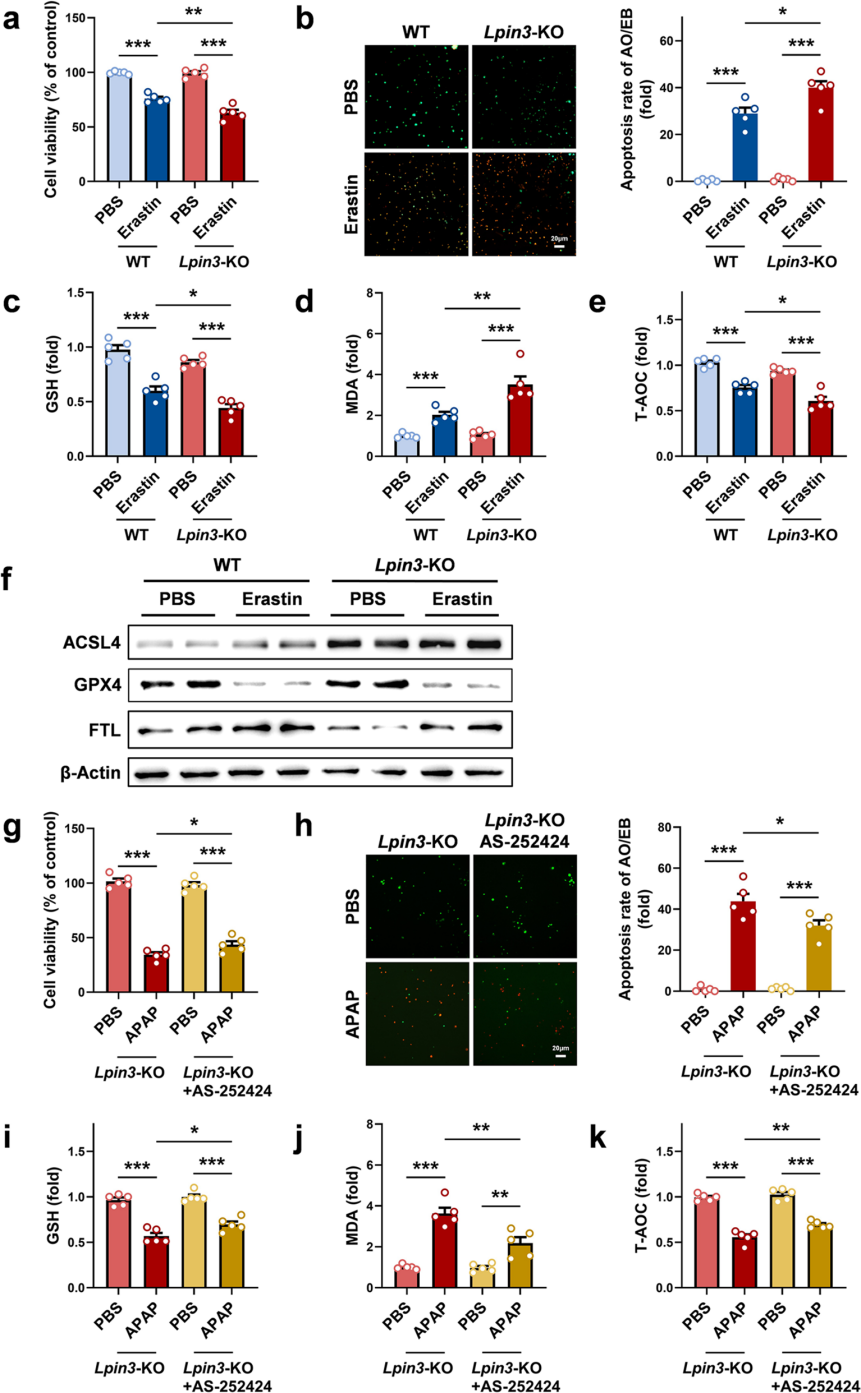
Lipin3 deficiency can activate hepatocytes ferroptosis. (**a**) CCK8 analysis showing the cell viability of WT and *Lpin3-*KO primary hepatocytes treated with or without Erastin. (**b**) AO/EB staining and statistical analysis of apoptosis rates in each primary hepatocytes group. (**c**) GSH levels, (**d**) MDA levels and (**e**) T-AOC levels in WT and *Lpin3*-KO primary hepatocytes treated with or without Erastin. (**f**) WB analysis revealed the expressions of ACSL4, FTL and GPX4 in WT and *Lpin3*-KO primary hepatocytes treated with or without Erastin. (**g**) CCK8 analysis showing the cell viability in *Lpin3*-KO primary hepatocytes treated with DMSO or AS-252424 followed by treating with or without APAP. (**h**) AO/EB staining and statistical analysis of apoptosis rates in each *Lpin3*-KO primary hepatocytes group. (**i**) GSH levels, (**j**) MDA levels and (**k**) T-AOC levels in *Lpin3-*KO primary hepatocytes treated with DMSO or AS-252424 followed by treating with or without APAP. *p < 0.05, **p < 0.01, ***p < 0.001

We further verified this through in vitro experiments. We stimulated primary hepatocytes from WT and *Lpin3-*KO mice with erastin, a ferroptosis inducer [20, 21]. CCK-8 assays revealed that erastin reduced cell viability, and the loss of cell viability was greater in *Lpin3*-KO cells than in WT cells (Fig. 4a). Similarly, AO/EB staining revealed that erastin stimulation caused cell death, with a significantly higher death rate in *Lpin3-*KO cells compared to WT cells (Fig. 4b). Biochemical analyses revealed that erastin treatment markedly reduced intracellular GSH levels and elevated MDA levels in primary hepatocytes. Compared with WT cells, *Lpin3*-KO cells exhibited exacerbated GSH depletion and a further increase in the level of MDA after APAP exposure (Fig. 4c and d). In addition, the T-AOC of *Lpin3*-KO cells was significantly lower than that of WT cells (Fig. 4e). These results suggested that *Lpin3-*KO cells are more susceptible to the toxicity of erastin. WB analysis revealed that the upregulation of ACSL4 expression in *Lpin3-*KO cells after erastin treatment was more pronounced than that in WT cells. Although erastin markedly reduced GPX4 levels, this reduction did not differ between genotypes. (Fig. 4f). To test therapeutic targeting, APAP-treated *Lpin3*-KO cells were incubated with the ACSL4 inhibitor AS-252424. The results of the CCK-8 assay revealed that APAP stimulation of *Lpin3*-KO cells led to a pronounced decrease in cell viability, which was significantly reversed by AS-252424 intervention (Fig. 4g). AO/EB staining results revealed that after APAP treatment, many *Lpin3*-KO cells died, whereas AS-252424 markedly reduced cell death (Fig. 4h). Biochemical assays revealed that APAP alone caused severe GSH depletion and marked MDA accumulation, and AS-252424 intervention significantly restored the GSH level while lowering the MDA content (Fig. 4i and j). Additionally, the APAP-induced reduction in T-AOC was effectively reversed by AS-252424 (Fig. 4k). Collectively, these data indicate that Lipin3 deficiency may exacerbate APAP-driven hepatocyte ferroptosis by increasing ACSL4 expression.

### Lipin3 deficiency activates the GSDME‑dependent pyroptosis in APAP induced ALI

**Fig. 5:**
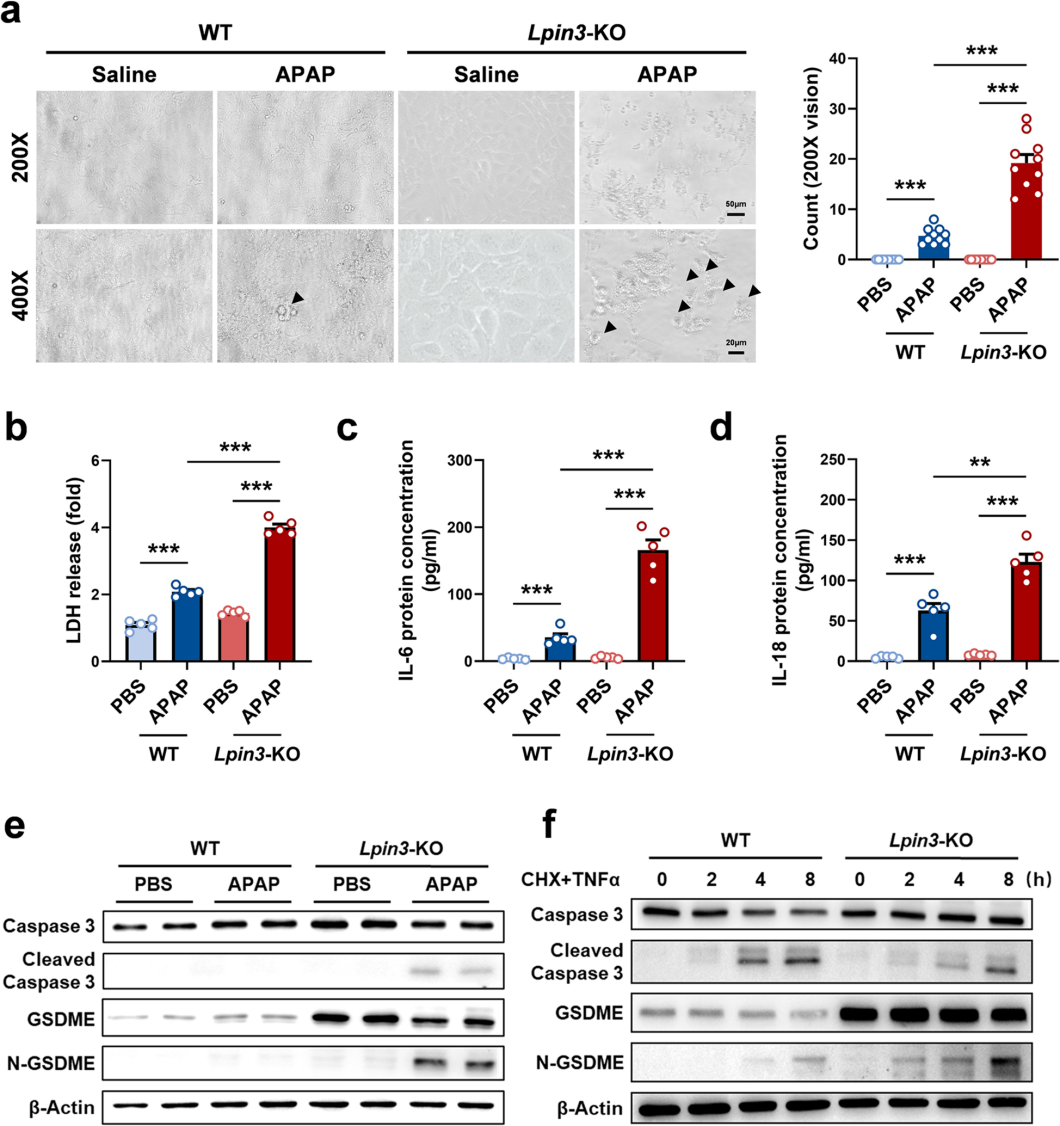
Lipin3 deficiency activate the GSDME‑dependent pyroptosis in APAP induced ALI. (**a**) Phase-contrast images of WT and *Lpin3-*KO primary hepatocytes were examined after APAP treatment. Arrows, pyroptotic cells. The levels of LDH (**b**), IL-6 (**c**) and IL-18 (**d**) in cell supernatant were measured. (**e**) WB analysis revealed the expressions of Caspase 3, Cleaved-Caspase 3, GSDME, N-GSDME in WT and *Lpin3-*KO primary hepatocytes treated with or without APAP. (**f**) WB analysis revealed the expressions of Caspase 3, cleaved-Caspase 3, GSDME and N-GSDME in WT and *Lpin3*-KO primary hepatocytes treated with CHX plus TNF for different lengths of time. **p < 0.01, ***p < 0.001

During cell processing, we observed the morphology of cell death under a light microscope and found that in *Lpin3-*KO cells, cell death increased after APAP treatment, and these cells presented morphological features such as swelling, blebbing, and a lantern-like appearance, which were not evident in the WT cells (Fig. 5a). A review of the literature revealed that such cell death morphology is characteristic of pyroptosis [22, 23]. Therefore, we collected the supernatants from each group of cells and measured the levels of lactate dehydrogenase (LDH) in the supernatants. APAP triggered a modest, non-significant rise in supernatant LDH from WT cells, whereas *Lpin3-*KO cells released markedly more LDH following the same stimulus (Fig. 5b). Additionally, the ELISA results also indicated that compared with those in the WT cells, the levels of the inflammatory factors interleukin-6 (IL-6) and interleukin-18 (IL-18) in the supernatants of *Lpin3-*KO cells significantly increased after APAP treatment (Fig. 5c and d). We collected proteins from each group of cells and detected the expression of pyroptosis-related proteins by WB. The results revealed that the pyroptosis-related Caspase 3-GSDME pathway was significantly activated in APAP-treated *Lpin3-*KO cells, as indicated by a significant increase in the cleavage of Caspase 3 and a significant increase in the level of activated N-GSDME (Fig. 5e). However, no significant changes in the expression of other pyroptosis-related proteins, such as GSDMA, GSDMB, GSDMC, or GSDMD, were detected (Figure S5). Notably, *Lpin3-*KO cells displayed markedly elevated baseline GSDME abundance compared with WT cells. The basal expression level of GSDME determines the degree of pyroptosis activation during the cell death process [22, 24]. Therefore, we switched to inducing Caspase 3 activation through a combination of cycloheximide (CHX) and tumor necrosis factor (TNF) stimulation [25, 26], to induce the same degree of Caspase 3 activation in both WT and *Lpin3-*KO cells, and observed the cleavage of GSDME. Compared with WT cells, *Lpin3-*KO cells with a higher basal level of GSDME exhibited greater N-GSDME cleavage (Fig. 5f). These results suggest that in APAP-induced ALI, Lipin3 deficiency can activate the pyroptosis of hepatocytes regulated by GSDME.

### Lipin3 can regulate ferroptosis and pyroptosis in hepatocytes via the JAK1-STAT3 pathway

**Fig. 6:**
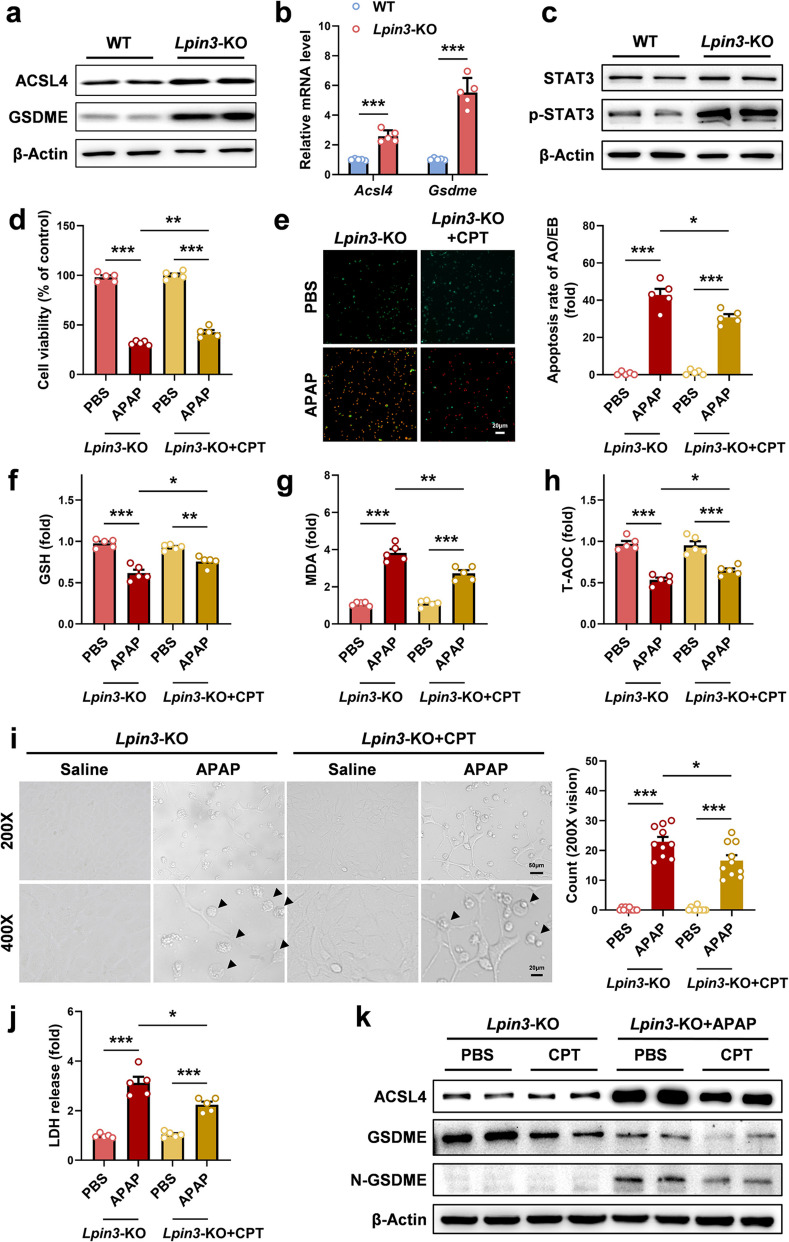
Lipin3 can regulate ferroptosis and pyroptosis via STAT3-ACSL4 and STAT3-GSDME pathway. (**a**) WB analysis revealed the expressions of ACSL4 and GSDME in WT and *Lpin3-*KO primary hepatocytes. (**b**) mRNA levels of *Acsl4* and *Gsdme* genes in WT and *Lpin3-*KO primary hepatocytes. (**c**) WB analysis revealed the expressions of STAT3 and p-STAT3 in WT and *Lpin3-*KO primary hepatocytes. (**d**) CCK8 analysis showing the cell viability in *Lpin3-*KO primary hepatocytes treated with DMSO or CPT followed by treating with or without APAP. (**e**) AO/EB staining and statistical analysis of apoptosis rates in each *Lpin3-*KO primary hepatocytes group. (**f**) GSH levels, (**g**) MDA levels and (**h**) T-AOC levels in *Lpin3-*KO primary hepatocytes treated with DMSO or CPT followed by treating with or without APAP. (**i**) Phase-contrast images of *Lpin3-*KO primary hepatocytes treated with DMSO or CPT followed by treating with or without APAP. Arrows, pyroptotic cells. (**j**) The levels of LDH in cell supernatant were measured. (**k**) WB analysis revealed the expressions of ACSL4, GSDME and N-GSDME in *Lpin3-*KO primary hepatocytes treated with DMSO or CPT followed by treating with or without APAP. *p < 0.05, **p < 0.01, ***p < 0.001

Previous results indicated that in APAP-induced ALI, the absence of Lipin3 significantly activated ACSL4-mediated ferroptosis and GSDME-mediated pyroptosis. Notably, compared with those in WT cells, the basal levels of ACSL4 and GSDME in *Lpin3-*KO cells were significantly greater (Fig. 6a). We hypothesized that Lipin3 levels might be associated with the expression of ACSL4 and GSDME, suggesting that Lipin3 could be involved in regulating the abundance of these proteins. Additionally, the qPCR results revealed that the mRNA levels of *Acsl4* and *Gsdme* were consistent with the changes in protein levels (Fig. 6b), indicating that the high levels of ACSL4 and GSDME in *Lpin3-*KO cells are regulated at the transcriptional level. Recent literature indicates that STAT3 is a common transcription factor for both ACSL4 and GSDME. p-STAT3 can regulate ACSL4-mediated ferroptosis and GSDME-mediated pyroptosis [27, 28]. WB detection of proteins from each group of cells revealed that compared with that in WT cells, the phosphorylation level of STAT3 in *Lpin3-*KO cells was significantly greater (Fig. 6c). We further selected APAP-treated *Lpin3-*KO primary hepatocytes and treated them with the STAT3 phosphorylation inhibitor cryptotanshinone (CPT) [29]. WB analysis confirmed that CPT markedly lowered STAT3 phosphorylation (Figure S6). The results of the CCK-8, AO/EB staining and biochemical assays revealed that APAP alone triggered pronounced loss of viability, cell death, GSH depletion, MDA accumulation, and reduced T-AOC, whereas CPT treatment significantly reversed these effects (Fig. 6d-h). In addition, CPT intervention markedly reduced APAP-induced pyroptotic cell counts and LDH release (Fig. 6i and j). Furthermore, WB results showed that CPT could significantly downregulate the abnormal upregulation and activation of ACSL4 and GSDME (Fig. 6k). Lipin3 can regulate STAT3 phosphorylation, which further affects the mRNA levels of ACSL4 and GSDME.

**Fig. 7:**
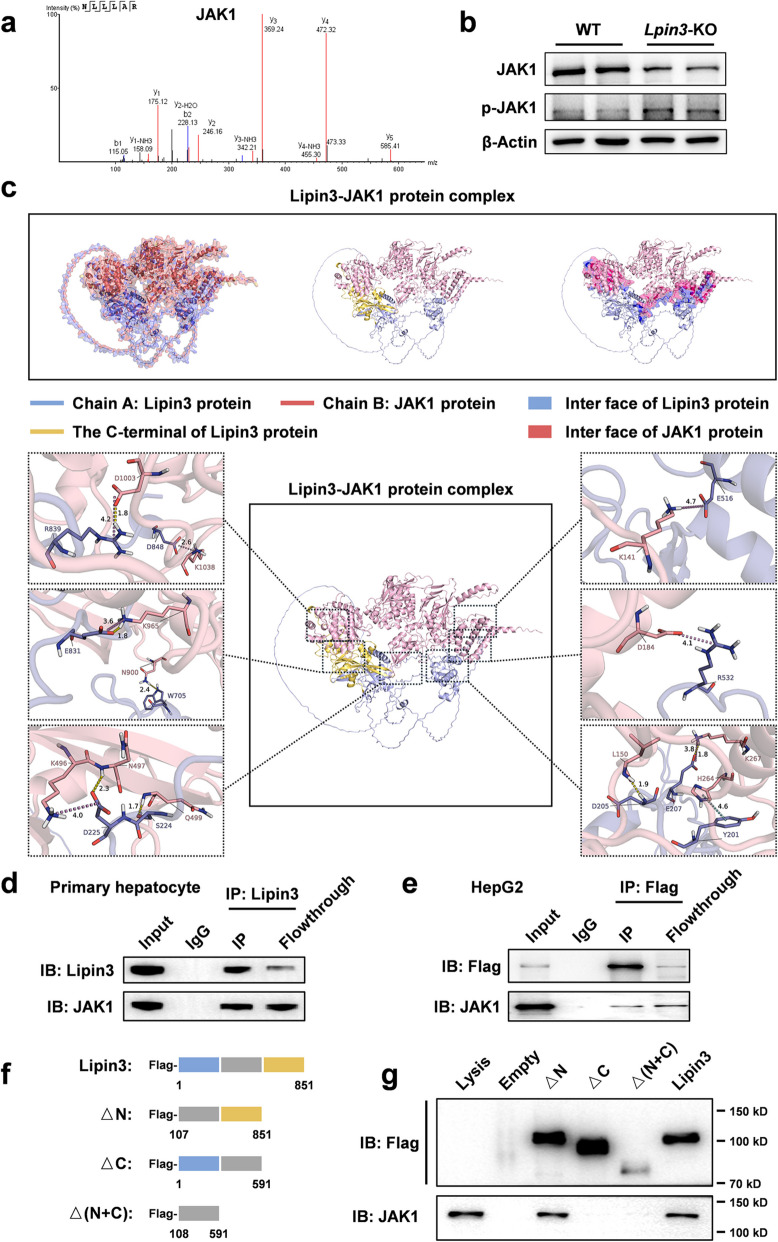
Lipin3 modulates STAT3 phosphorylation by interacting with JAK1. (**a**) Mass spectrometry results indicate that JAK1 is a potential interacting protein of Lipin3. (**b**) WB analysis revealed the expressions of JAK1 and p-JAK1 in WT and *Lpin3-*KO primary hepatocytes. (**c**) Flexible docking predictions reveal the JAK1-Lpin3 interaction interface, identifying multiple binding sites between the two proteins. Co-IP analysis confirmed the interaction between Lipin3 and JAK1 in primary hepatocytes (**d**) and HepG2 cells (**e**, **f**) Schematics depicting Flag-Lpin3 fusion proteins lacking the N-terminus (△N), the C-terminus (△C), or both the N-terminus and C-terminus (△N + C) are shown. (**g**) Co-IP analysis showed the interaction of Flag-Lpin3 fusion proteins missing the N-terminus, C-terminus, or both interact with JAK1

Interestingly, through mass spectrometry experiments, we identified JAK1 as a candidate protein that interacts with Lipin3 (Fig. 7a). JAK1 is upstream of STAT3 and regulates the phosphorylation of STAT3. WB analysis also confirmed that, compared with that in WT cells, the phosphorylation level of JAK1 was significantly elevated in *Lpin3-*KO cells (Fig. 7b). Using AlphaFold3, we performed five flexible docking simulations for the Lipin3–JAK1 complex (Figure S7). The results of bioinformatics software revealed multiple interaction interfaces, with Lipin3’s binding regions predominantly located at its C-terminus. Ranked by binding energy, the top-scoring model was selected to delineate the putative Lipin3-JAK1 interaction sites (Fig. 7c and Table S2). Co-IP assays in primary hepatocytes and HepG2 cells confirmed the interaction between Lipin3 and JAK1 (Fig. 7d and e). We further constructed plasmids encoding full-length Lipin3, Lipin3 with an N-terminal deletion (△N), Lipin3 with a C-terminal deletion (△C), and Lipin3 with both N- and C-terminal deletions (△N + C) (Fig. 7f). These plasmids were transfected into HepG2 cells, and co-IP and WB experiments were performed to explore the binding regions of the JAK1 and Lipin3 proteins. When the C-terminus was deleted, Lipin3 lost its ability to bind to the JAK1 protein (Fig. 7g). The C-terminus of Lipin3 contains a key HAD phosphatase motif, DXDXT, which mainly functions to mediate the activity of the phospholipid phosphatase PAP [15]. Therefore, we speculate that under normal conditions, Lipin3 can interact with JAK1 to mediate its dephosphorylation of JAK1. When Lipin3 is deficient, it cannot interact with JAK1, and the dephosphorylation of JAK1 is weakened, leading to abnormal upregulation of JAK1 phosphorylation. Abnormally upregulated p-JAK1 leads to abnormal upregulation of p-STAT3, inducing the transcriptional upregulation of downstream ACSL4 and GSDME and the overactivation of ferroptosis and pyroptosis, ultimately exacerbating liver injury.

### Overexpression of Lipin3 alleviated hepatocyte ferroptosis and pyroptosis in APAP induced ALI

**Fig. 8:**
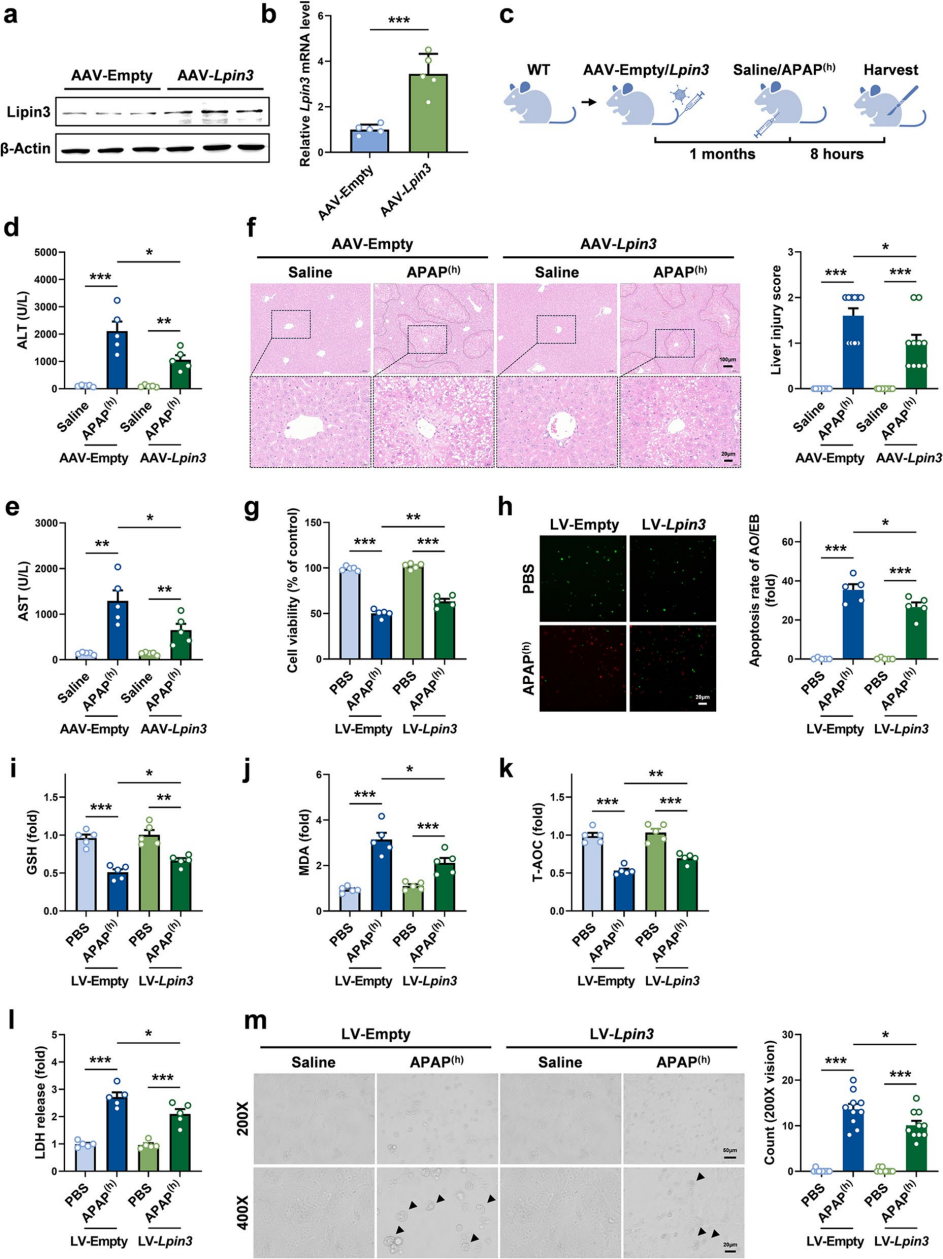
Increasing Lipin3 can alleviate APAP-induced ALI. WB analysis (**a**) and qPCR (**b**) confirmed successful overexpression of Lipin3 in the mice liver. (**c**) Flow chart of animal study design. Peripheral blood ALT (**d**) and AST (**e**) levels of AAV-Empty mice (*n* = 5) and AAV-*Lpin3* mice (*n* = 5) treated with or without high-dose APAP. (**f**) HE staining analysis showing the liver injury in AAV-Empty mice (*n* = 5) and AAV-*Lpin3* mice (*n* = 5) treated with or without high-dose APAP. (**g**) CCK8 analysis showing the cell viability in WT primary hepatocytes treated with LV-Empty or LV-*Lpin3* infection followed by treating with or without high-dose APAP. (**h**) AO/EB staining and statistical analysis of apoptosis rates in each WT primary hepatocytes group. (**i**) GSH levels, (**j**) MDA levels and (**k**) T-AOC levels in WT primary hepatocytes treated with LV-Empty or LV-*Lpin3* infection followed by treating with or without high-dose APAP. (**l**) The levels of LDH in cell supernatant were measured. (**m**) Phase-contrast images of WT primary hepatocytes infected with LV-Empty or LV-*Lpin3* followed by treating with or without high-dose APAP. Arrows, pyroptotic cells. *p < 0.05, **p < 0.01, ***p < 0.001

Finally, we investigated whether increased Lipin3 expression can alleviate APAP-induced ALI. Lipin3 overexpression was established in 8-week-old WT mice via a single tail-vein administration of AAV (Fig. 8a and b). We induced more severe ALI in mice by intraperitoneal injection of a high dose of APAP. After the modeling was completed, the mice were sacrificed, and samples were taken for subsequent detection (Fig. 8c). Biochemical analysis revealed that, after APAP treatment, circulating ALT and AST were markedly lower in AAV-*Lpin3* mice than in AAV-Empty mice (Fig. 8d and e). Moreover, the HE staining results revealed that compared with that in AAV-Empty mice treated with APAP, liver injury in AAV-*Lpin3* mice was significantly reduced (Fig. 8f). These results indicate that overexpression of Lipin3 can alleviate APAP-induced ALI. Next, we selected WT primary hepatocytes treated with high-dose APAP and overexpressing *Lpin3* through lentivirus infection. The results of the CCK-8, AO/EB staining and biochemical assays revealed that high-dose APAP alone triggered pronounced loss of viability, cell death, GSH depletion, MDA accumulation and reduced T-AOC, whereas *Lpin3* overexpression significantly reversed these effects (Fig. 8g-k). In addition, *Lpin3* overexpression markedly reduced high-dose APAP-induced pyroptotic cell counts and LDH release (Fig. 8l and m). These results indicate that increasing Lipin3 levels in ALI can alleviate APAP-induced hepatocellular ferroptosis and pyroptosis.

Collectively, our data suggest that Lipin3 can interact with JAK1 to dephosphorylate p-JAK1. Lipin3 deficiency can lead to elevated levels of p-JAK1, which in turn activates the downstream p-STAT3-ACSL4 and p-STAT3-GSDME pathways. This exacerbates ferroptosis and pyroptosis in hepatocytes induced by APAP, ultimately worsening the progression of ALI (Figure S8).

## Discussion

As an antipyretic, APAP was widely used to treat fever caused by the Coronavirus disease 2019 (COVID-19) during the pandemic [30]. However, the side effects of APAP can lead to ALI, which increases the risk of death among COVID-19 patients [31]. Therefore, identifying more targets that are sensitive to APAP toxicity can help guide clinical medication, reduce liver damage caused by APAP, and increase the safety of treatment for COVID-19 patients. Our study revealed that serum Lipin3 concentrations in ALI patients decreased in direct proportion to the extent of hepatic damage, indicating that lower Lipin3 levels are associated with more severe liver injury. Additionally, compared with WT mice, *Lpin3-*KO mice exhibited more severe liver injury after the administration of APAP. In vitro experiments also confirmed that Lipin3 knockout increases cellular sensitivity to APAP-induced toxicity. Our results suggested that Lipin3 might be a novel candidate target for APAP toxicity sensitivity. Therefore, we recommend that individuals with low Lipin3 expression should exercise caution when considering APAP as an antipyretic.

The Lipin protein family is crucial for lipid metabolism and hepatic lipid homeostasis. Recent studies have explored their roles in liver diseases. Lipin1 plays a significant role in the pathogenesis of alcoholic fatty liver disease, and its liver expression is correlated with metabolic dysfunction-associated fatty liver disease severity [32, 33]. Reduced Lipin1 levels correlate with both insulin resistance and hepatic steatosis progression [34, 35]. Research has also shown that Lipin2 acts as a key bridge between endoplasmic reticulum stress and hepatic insulin resistance [36]. Both Lipin1 and Lipin2 are involved in the regulation of the hepatitis C virus (HCV) replication cycle, suggesting that they play regulatory roles in HCV-infected livers [37, 38]. Lipin3 is the least studied member of the Lipin family, but many mechanisms remain unclear. Our previous research revealed that haploinsufficiency of Lipin3 may disrupt the nucleocytoplasmic distribution of Lipin1, leading to abnormal lipid metabolism and fatty liver [19]. However, these studies focused on lipid metabolism-related liver diseases. In acute liver diseases, such as APAP-induced ALI, the role of Lipin family members has not been explored. Therefore, our study investigated the role of Lipin3 in APAP-induced ALI and revealed that Lipin3 deficiency increases the sensitivity of the body to APAP drug toxicity, leading to severe liver injury. Our research expands the current understanding of Lipin3 and fills a gap in this field.

Pyroptosis has recently garnered widespread attention because of its role in liver injury. Hepatocyte pyroptosis is activated in various models of liver injury, including drug-induced liver injury, alcoholic liver disease (ALD), and nonalcoholic steatohepatitis (NASH) [39]. In ALD, excessive alcohol consumption induces the activation of Caspase-4/11, which cleaves GSDMD to form pores in the plasma membrane, triggering pyroptosis and thereby promoting the progression of ALD [39, 40]. In NASH, ceramides associated with lipotoxicity can induce the assembly of the NLRP3 inflammasome, activating Caspase-1 to cleave GSDMD and ultimately inducing hepatocyte pyroptosis [41]. GSDMD plays a crucial role in the progression of NASH by regulating cytokine secretion, NF-κB activation, and lipogenesis [42]. Deoxynivalenol can induce inflammatory responses in mouse livers and HepG2 cells, activating pyroptosis mediated by the Caspase-3/GSDME pathway [43]. In a sepsis mouse model, inhibiting GSDME-mediated pyroptosis alleviated liver injury induced by cecal ligation and puncture [44]. Additionally, activation of GSDME in mouse models and patient liver tissues with APAP-induced ALI has been reported. In mice, knockout of GSDME, but not GSDMD, protects against APAP-induced liver injury, whereas hepatocyte-specific restoration of GSDME recapitulates APAP-induced liver injury [14]. Despite these findings, the precise regulatory mechanisms of hepatocyte pyroptosis in liver injury remain to be fully elucidated and require further investigation. Our study revealed that Lipin3 plays a significant regulatory role in APAP-induced ALI, potentially through modulating pyroptosis, thereby providing new insights for research in this area.

The molecular mechanisms of ferroptosis are strongly associated with APAP-induced liver injury. In APAP-induced liver injury, the excessive metabolic product NAPQI depletes GSH and disrupts GPX4 function, leading to mitochondrial lipid peroxidation and loss of membrane integrity [5]. In mouse models treated with APAP, the expression of markers of ferroptosis (such as MDA and 4-hydroxynonenal) is significantly elevated [45]. Mass spectrometry analysis revealed that the levels of lipid peroxidation products derived from n-6 fatty acids significantly increase in the livers of APAP-treated mice and that the accumulation of these products is closely related to mitochondrial membrane damage. The inhibition of ACSL4 (the key enzyme regulating the incorporation of polyunsaturated fatty acids into membrane phospholipids) or supplementation with α-tocopherol (a lipid antioxidant) can effectively block APAP-induced ferroptosis and liver injury [46, 47]. Although the role of ferroptosis in APAP-induced liver injury has been supported by several studies, its pathological significance remains controversial. Some studies have shown that the standard ferroptosis inhibitor Fer-1 does not completely improve liver injury or reduce lipid peroxidation levels in APAP models without additional iron loading, suggesting that ferroptosis may not be the sole mechanism of APAP toxicity. The contribution of ferroptosis may depend on experimental conditions (such as iron overload status) or interactions with other death pathways (such as pyroptosis and necroptosis) [45, 48]. For example, the release of IL-1β after NLRP3 inflammasome activation can exacerbate oxidative stress and promote ferroptosis. Conversely, damage-associated molecular patterns released by ferroptosis may also activate the pyroptosis pathway [49]. Similarly, we demonstrated that Lipin3 deficiency significantly exacerbated APAP-induced liver injury, accompanied by significant activation of pyroptosis and ferroptosis. Mechanistic exploration revealed that ACSL4 was significantly activated in Lipin3-deficient cells after APAP treatment, which may partially explain the abnormal activation of ferroptosis. However, we did not comprehensively detect all ferroptosis-related pathways. Our results highlight the significant contribution of ferroptosis to APAP-induced liver injury and suggest that ferroptosis may coexist with and interact with other cell death pathways.

The JAK1-STAT3 pathway is involved in ALI, and its inhibition can protect against liver damage. For example, the JAK1 inhibitor upadacitinib reduces hepatocyte lipid accumulation and apoptosis [50]. Graveoline, an alkaloid from Mosla chinensis, mitigates lipopolysaccharide-induced liver injury by inhibiting JAK1 and STAT3 phosphorylation [51]. In fenitrothion poisoning, saponarin downregulates the JAK1-STAT3 pathway, alleviating hepatotoxicity [52]. Collectively, these findings underscore the JAK1–STAT3 axis as both a driver of ALI and a promising therapeutic focus. Our study revealed that Lipin3 can modulate the JAK1-STAT3 pathway and may act as a promising therapeutic target in APAP-induced ALI. The JAK1-STAT3 signaling pathway orchestrates both inflammatory signaling and programmed cell death. STAT3, acting as a transcription factor, can directly or indirectly regulate multiple genes associated with ferroptosis, such as *GPX4*, *SLC7A11*, and *ACSL4*, thereby influencing cellular sensitivity to ferroptosis [27, 53, 54]. Recent studies have confirmed that STAT3 can also affect cell fate by regulating the expression of genes related to pyroptosis. In glioblastoma cells, STAT3 is involved in regulating pyroptosis mediated by GSDMD [55]. Moreover, research by Wei et al. has confirmed that STAT3 is directly related to and positively regulates the expression of GSDME, which acts as a transcription factor [28]. These studies suggest that STAT3 may regulate multiple cell death pathways. Wu et al. linked ginsenoside Rh3 to Stat3-driven pyroptosis and ferroptosis in colorectal cancer [56]. Similarly, we demonstrated that Lipin3 can modulate GSDME and ACSL4 via the JAK1–STAT3 pathway, thereby regulating both death modes in APAP-induced ALI. These findings further broaden the understanding in this area.

In this study, we revealed that Lipin3 expression is significantly downregulated in an APAP-induced ALI model and that Lipin3 deficiency exacerbates the ALI phenotype. However, the precise mechanisms underlying APAP-induced Lipin3 downregulation remain to be elucidated. We also discovered that overexpressing Lipin3 markedly mitigated APAP-induced hepatocyte ferroptosis and pyroptosis, thereby alleviating the ALI phenotype. Despite the therapeutic potential of Lipin3 highlighted by these findings, we have not yet developed small-molecule drugs targeting Lipin3 for ALI treatment. These challenges will form the paramount focus of our subsequent investigations. Lipin3, as a potential regulatory target, warrants further in-depth investigation in terms of its role in pyroptosis and ferroptosis, with the aim of providing new therapeutic strategies for APAP-induced liver injury.

In conclusion, our study underscores the essential protective function of Lipin3 in the liver against ALI. Lipin3 deficiency can trigger GSDME-mediated pyroptosis and ACSL4-mediated ferroptosis via the JAK1-STAT3 signaling pathway in hepatocytes, thereby exacerbating ALI induced by APAP. Enhancing Lipin3 expression could be a viable therapeutic avenue for ameliorating ALI.

## Materials and methods

### Human samples, mouse strain and cell lines

This research was reviewed and approved by the Institutional Review Board Committee of Xiangya Hospital, Central South University, China. The committee granted approval for the human subjects component under protocol number 2024–1-14, and for the animal studies under protocol number 2024–2-15. Written informed consent was obtained from all human participants involved in the study.

*Lpin3* knockout (*Lpin3*-KO) mice, in which exons 3–7 were deleted in the C57BL/6 J background, have been described previously [19]. Wild-type (WT) mice (C57BL/6 J background) were acquired from Cyagen Company (SuZhou, China). Lipin3 was overexpressed in vivo via tail vein injection of AAV9-*Lpin3* (1 × 10^12^ copies/mouse; pCMV promoter; GeneChem Co., Ltd), with AAV9-Empty as control. [57, 58]. Four weeks post-injection, successful overexpression was confirmed by Western blotting. To establish APAP-induced AlI models in mice, 10-week-old WT and Lpin3-KO mice were injected with 100 mg/kg APAP. In the overexpression experiments, Lipin3-overexpressing mice were injected with a high dose of APAP (300 mg/kg). The mice were euthanized 8 h post-injection, and serum along with liver samples were harvested for subsequent analysis.

The commercial HepG2 cell line was acquired from the Cell Bank of Shanghai Institutes for Biological Sciences (China). Mouse primary hepatocytes were isolated using a previously established protocol [19]. In the in vitro overexpression assay, primary hepatocytes were transfected with lentivirus (LV). Primary hepatocytes (1 × 10^5^) were transduced with 2 × 10⁶ TU of LV-Empty or LV-*Lpin3* lentivirus, following the standardized protocol provided by GeneChem (Shanghai, China). Both the HepG2 cell line and primary hepatocytes were maintained in DMEM/F-12 medium supplemented with 10% fetal bovine serum, 100 U/ml penicillin, and 100 μg/ml streptomycin at 37 °C under 5% CO₂. In vitro, the cells were treated with 20 μM APAP for 12 or 24 h. In the overexpression experiments, the cells were treated with a high dose of APAP (40 μM).

### Key reagents

The Lipin3 antibody used in this study was generated in our laboratory as we previously described [19]. Antibodies against β-actin (Cat No. 20536–1-AP), STAT3 (Cat No: 60199) were purchased from Proteintech Group, Inc. Antibodies against GSDME (# 19453S), gasdermin A (# 49307S), gasdermin B (# 64436 T), gasdermin C (# 61921 T), gasdermin D (# 32112 T), Caspase 3 (# 9662), p-STAT3 (# 9145S) and JAK1 (# 3332S) were purchased from Cell Signaling Technology. Antibodies against ACSL4 (sc-271800), p-JAK1 (sc-1677) and FTL (sc-390558) were obtained from Santa Cruz Biotechnology. Other antibodies used were those against Flag (ab205606; Abcam), HMGB1 (ET1601-2; HuaBio, China), and GPX4 (R381958; Zhengneng Biotechnology Co., Ltd., ZEN-BIO). The Hematoxylin–Eosin/HE Staining Kit (G1120), Glutamic-pyruvic Transaminase (ALT) Assay Kit (BC1550), Glutamic-oxalacetic Transaminase (AST) Assay Kit (BC1560), Broad-Spectrum Immunohistochemistry Kit (SP0041) and Acridine Orange (AO)/EB Double Staining Kit (CA1140-100) were obtained from Beijing Solarbio Science & Technology Co., Ltd. The Cell Counting Kit-8 (C0037), TUNEL Apoptosis Assay Kit (C1086), LDH Release Assay Kit (C0016) and Protein A + G beads (P2108) were obtained from Beyotime Biotechnology. A mouse IL-6 ELISA kit (JL20268) and a mouse IL-18 ELISA kit (JL20253) were obtained from Jianglai Biology. A human Lipin3 ELISA detection kit (LZ-E031454) was purchased from Shanghai Rayzbio Biotechnology Co., Ltd. APAP (HY-66005) was purchased from MedChemExpress. Erastin (KM9100), AS-252424 (KM7417) and CPT (KM15658) were purchased from KKLmed, INC, USA. An MDA content assay kit (AKFA013M), a GSH content assay kit (AKPR008M) and a T-AOC assay kit (AKAO012M) were purchased from Beijing Boxbio Science & Technology Co., Ltd.

### Data source

Gene expression datasets were acquired from the Gene Expression Omnibus (GEO) database (http://www.ncbi.nlm.nih.gov/geo/) using the search terms “ALI”, “APAP” and “Expression profiling by array”. After screening, datasets GSE275008, GSE104601, GSE241511 and GSE230831 were identified and subjected to subsequent bioinformatics analysis.

### Western blot (WB)

Proteins were extracted from cells or tissues using RIPA lysis buffer and quantified with a Pierce™ BCA kit (23,225; Thermo Fisher Scientific). For electrophoresis, 30 μg of protein per sample was resolved on Bis–Tris NuPAGE gels (4%–12%; EC6026BOX, Invitrogen™). Following incubation with primary and secondary antibodies, protein bands were visualized using an Alpha Innotech chemiluminescence imaging system.

### HE staining, immunohistochemistry (IHC), and TUNEL staining

Standard protocols were employed for the preparation of 6-μm sections from paraformaldehyde-fixed, paraffin-embedded liver tissues. HE and immunohistochemical (broad-spectrum kit) staining were performed and evaluated by light microscopy. TUNEL apoptosis assay was conducted and sections were imaged using a Leica DM500 fluorescence microscope.

### AST, ALT and LDH detection

Levels of ALT and AST in serum and LDH in cell culture medium were measured in supernatants (centrifuged at 1000 × g, 5 min, 4 °C) using specific assay kits and a Cary 60 UV–Vis colorimeter (Agilent), with detection at 505 nm for transaminases and 600 nm for LDH.

### CCK8, flow cytometric analysis and AO/EB staining

Cell viability was determined using a Cell Counting Kit-8 (CCK-8) assay. Briefly, cells were seeded in 96-well plates at 5,000 cells/well. After treatment, CCK-8 reagent was added directly to the culture medium and incubated for 1 h at 37 °C. Absorbance was measured at 450 nm using a Bio-Rad microplate reader.

After treatment with APAP in 25 cm^2^ dishes, cells were subjected to annexin V/PI staining using a solution containing 5 μl annexin V-FITC and 10 μl PI in 195 μl binding buffer. Following 15 min of dark incubation, apoptosis was quantified by flow cytometry (BD FACSCanto).

For AO/EB staining, cells grown on slides were fixed with 4% paraformaldehyde and permeabilized with 0.5% Triton X-100. Subsequently, the cells were stained using an AO/EB double-staining kit and visualized under a Leica SP5 microscope following standard protocols.

### Molecular docking simulations

The AlphaFold3 online platform (https://alphafoldserver.com/) was employed for flexible molecular docking simulations. PRODIGY software was used to predict the binding affinity in protein–protein complexes [59]. PyMOL was used to create high-quality visualizations of the biomolecular structures.

### Plasmid construction and transfection

A wild-type (WT) Lipin3 CDS containing a C-terminal Flag-tag was first generated in the pEnter vector. Subsequently, plasmids encoding full-length Lipin3, Lipin3 with an N-terminal deletion (△N), Lipin3 with a C-terminal deletion (△C), and Lipin3 with both N- and C-terminal deletions (△N + C) were constructed. These plasmids were transiently transfected using Lipofectamine™ 2000 CD (Thermo Fisher Scientific), in accordance with the manufacturer’s protocol.

### RNA-seq and real-time polymerase chain reaction (PCR)

Total RNA was isolated from tissues using the PureLink® RNA Mini Kit and reverse-transcribed into cDNA with an All-in-One First-Strand Synthesis Master Mix (Yugong Biotech). Gene expression was assessed by qRT-PCR with F488 SYBR qPCR Mix (Universal; BestEnzymes, EG23111L) and normalized to controls using the 2^(−△△Ct)^ method, with all experiments conducted in triplicate. Primer sequences are provided in Table S3.

### Mass spectrometry analysis and coimmunoprecipitation (co-IP)

Tissue lysates from WT mice (500 μg in 1 mL) were subjected to overnight immunoprecipitation at 4 °C with Protein A + G beads (Beyotime, P2108), using either IgG or a Lipin3-specific antibody. The potential interacting proteins were extracted at 10,000 × *g* for 10 min at 4 °C. For mass spectrometric analysis, the extensively washed immunoprecipitates were further analyzed by an Orbitrap Eclipse Tribrid mass spectrometer (Thermo Fisher Scientific) at the Center for Advanced Studies, Central South University.

For co-IP analysis, the immunoprecipitates were thoroughly washed, resolved on a 4–12% NuPage Bis–Tris gel, and subsequently subjected to Western blotting with the indicated antibodies. Chemiluminescent signals were captured and quantified based on integrated density values using an Alpha Innotech imaging system.

### Statistical analysis

All data were evaluated in GraphPad Prism and are shown as mean ± SEM from at least three independent experiments. For two-group comparisons, a two-tailed Student’s t-tests based on ANOVA was applied. Significance was defined as *p* < 0.05, with asterisks denoting *p < 0.05, **p < 0.01, ***p < 0.001; ns represents not significant.

## Supplementary Information

Supplementary Material 1

## Data Availability

The bioinformatics data supporting the findings of this study are openly available in the Gene Expression Omnibus under accession GSE275008, GSE104601, GSE241511 and GSE230831. Additional experimental protocols, reagent information, and additional data are available within the article and/or its supplementary materials.
