# Supplementary material for: Lipin3 deficiency promotes hepatocyte ferroptosis and pyroptosis via activating JAK1-STAT3 pathway during acetaminophen induced acute liver injury
Source: Mol Biomed. 2025 Oct 10;6:78. doi: 10.1186/s43556-025-00317-z (PMC12514125; doi:10.1186/s43556-025-00317-z)
Supplement: Supplementary file 1 — Supplementary Material 1 [file 43556_2025_317_MOESM1_ESM.docx]

Lipin3 deficiency promotes hepatocyte ferroptosis and pyroptosis via activating JAK1-STAT3 pathway during acetaminophen induced acute liver injury

Yu-Xing Liu^1,2#^, Qian Wang^1#^, Zi-Yu Xiangyang^1^, Jie-Yi Long^1^, Hao Huang^1,2^, Liang-Liang Fan^1*^

^1^. Department of Cell biology, School of Life Science, Central South University, Changsha, 410013, China

^2^. Department of Nephrology, Xiangya Hospital of Central South University,

Changsha, 410028, China

# These authors contributed equally to this work.

* Correspondence:

Dr. Liang-Liang Fan

E-mail: [swfanliangliang@csu.edu.cn](mailto:swfanliangliang@csu.edu.cn)

Department of Cell biology, School of Life Science, Central South University, Changsha, 410013, China.


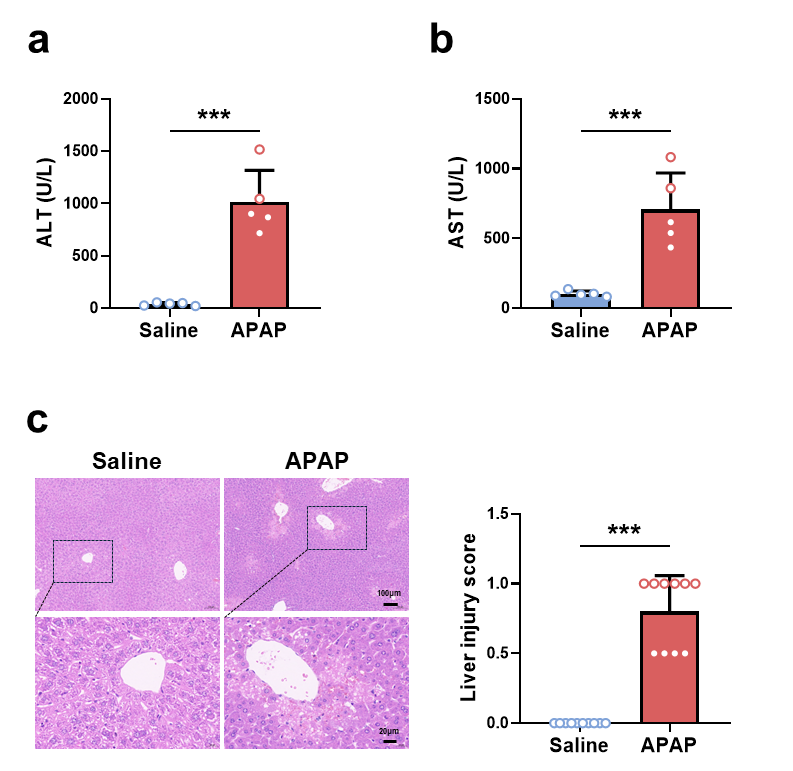


Supplementary Figure 1. Peripheral blood ALT (a) and AST (b) levels of WT mice (n = 5) treated with or without APAP. (c) HE staining analysis showing the liver injury in WT mice (n = 5) treated with or without APAP. ****p* < 0.001.

Supplementary Figure 2. The expression of Lipin3 in the peripheral serum of healthy individuals and APAP-ALI patients. **p* < 0.05.


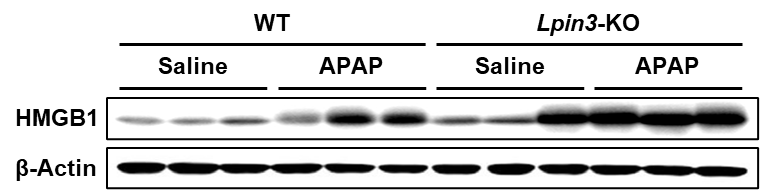


Supplementary Figure 3. WB analysis revealed the expression of HMGB1 in WT mice and *Lpin3*-KO mice treated with or without APAP.


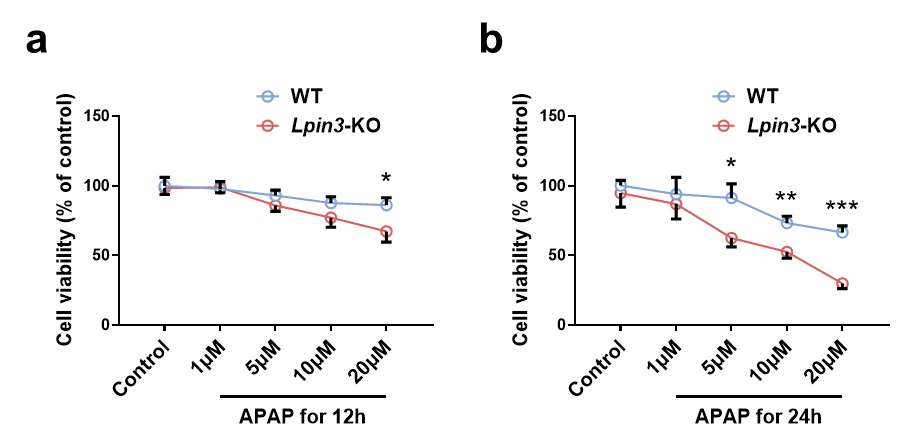


Supplementary Figure 4. CCK8 analysis showing the cell viability of WT and *Lpin3*-KO primary hepatocytes treated with different concentrations of APAP for 12h (a) or 24h (b). **p* < 0.05, ***p* < 0.01, ****p* < 0.001.


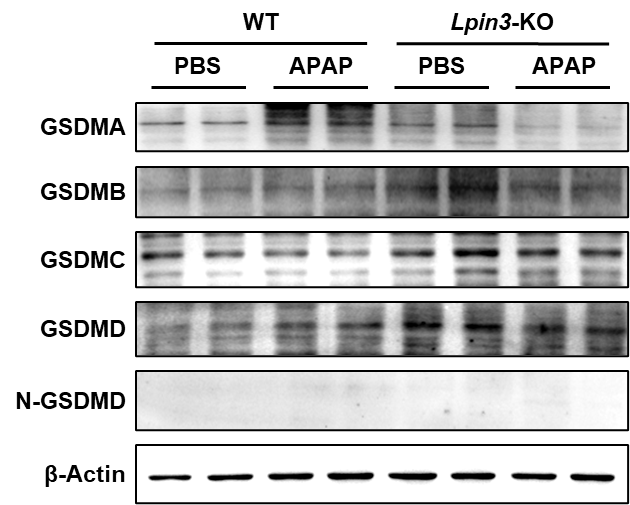


Supplementary Figure 5. WB analysis revealed the expressions of GSDMA, GSDMB, GSDMC, GSDMD and N-GSDMD in WT and *Lpin3*-KO primary hepatocytes treated with or without APAP.


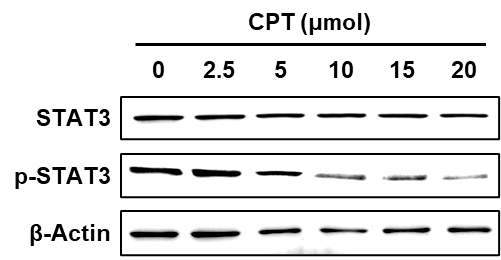


Supplementary Figure 6. WB analysis revealed the expressions of STAT3 and p-STAT3 in *Lpin3*-KO primary hepatocytes treated with CPT for different concentration.


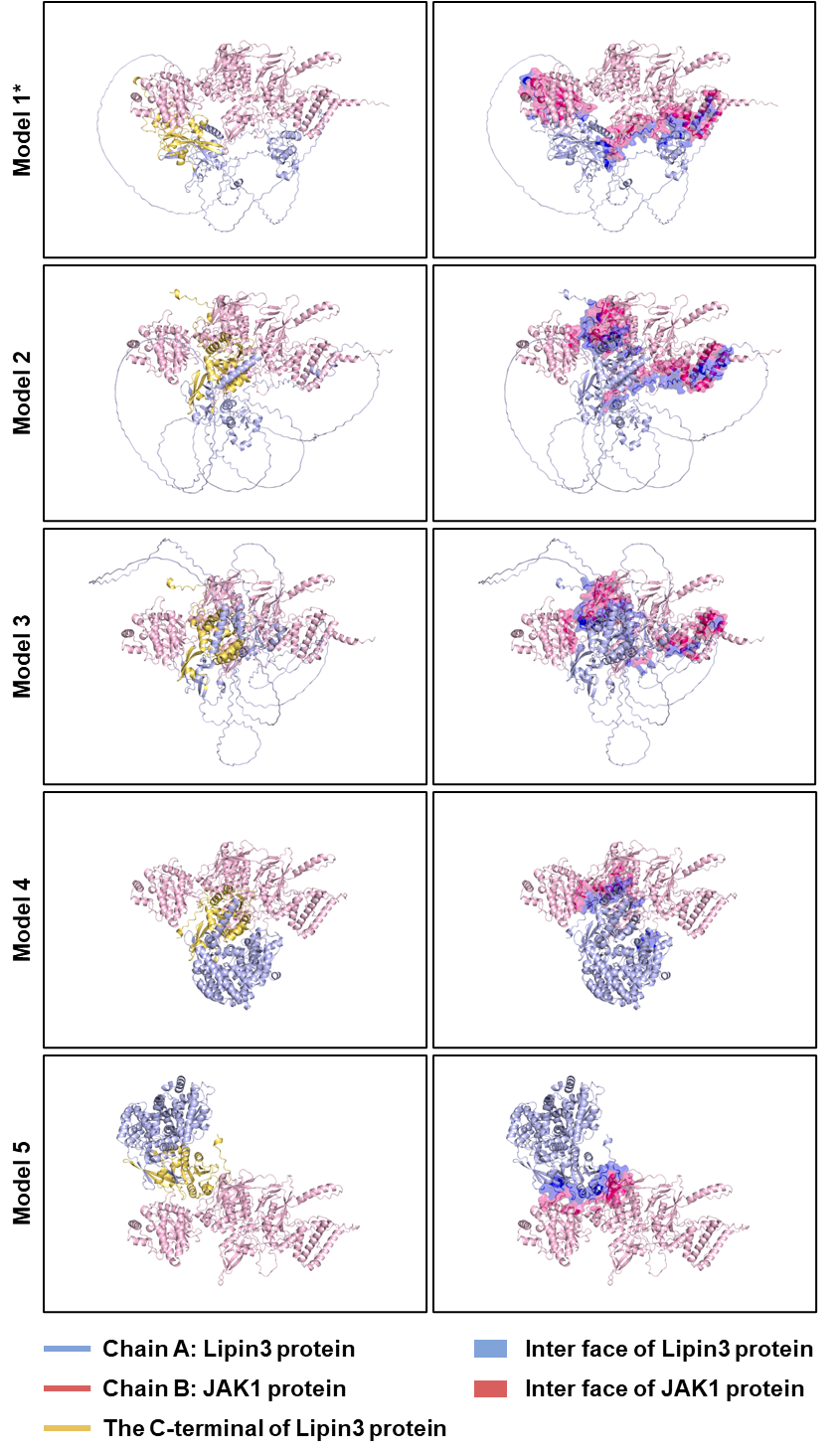


Supplementary Figure 7. AlphaFold3 generated five possible flexible docking models for the interaction between Lipin3 and JAK1.


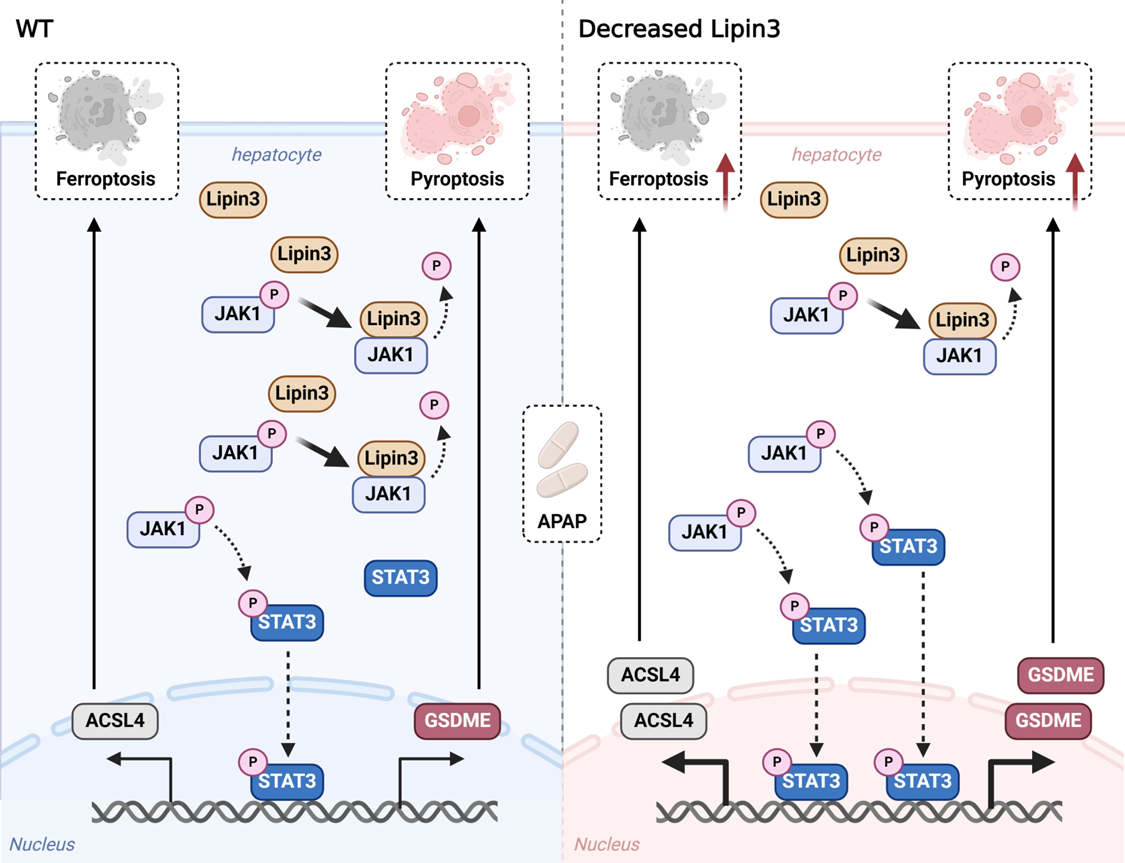


Supplementary Figure 8. The mechanism between Lipin3 and APAP-induced ALI. The figure was created with BioRender.com.

Table S1. Clinical characteristics of 36 patients.

| Patient | Age | Sex | ALT (U/L) | AST (U/L) | ALI etiology |
| --- | --- | --- | --- | --- | --- |
| 1 | 58 | M | 252.5 | 356.10 | Drug-induced |
| 2 | 59 | F | 568.1 | 849.10 | Drug-induced (APAP) |
| 3 | 39 | M | 190.9 | 177.70 | Infectious |
| 4 | 30 | M | 351.0 | 220.00 | Drug-induced (APAP) |
| 5 | 66 | M | 314.0 | 251.10 | Drug-induced |
| 6 | 55 | M | 235.1 | 221.10 | Infectious |
| 7 | 42 | F | 682.0 | 460.00 | Drug-induced (APAP) |
| 8 | 47 | M | 503.1 | 400.00 | Infectious |
| 9 | 60 | M | 250.2 | 222.00 | Toxic |
| 10 | 50 | M | 249.0 | 242.00 | Ischemic |
| 11 | 56 | F | 610.0 | 804.00 | Ischemic |
| 12 | 33 | F | 186.2 | 225.60 | Infectious |
| 13 | 27 | M | 539.0 | 700.00 | Drug-induced (APAP) |
| 14 | 36 | M | 487.6 | 396.60 | Infectious |
| 15 | 51 | M | 193.6 | 464.10 | Drug-induced |
| 16 | 51 | M | 171.9 | 213.00 | Drug-induced |
| 17 | 59 | M | 213.6 | 200.20 | Drug-induced |
| 18 | 23 | M | 519.0 | 741.00 | Drug-induced (APAP) |
| 19 | 38 | F | 280.0 | 290.00 | Drug-induced |
| 20 | 33 | M | 269.0 | 195.00 | Drug-induced |
| 21 | 31 | F | 167.4 | 182.20 | Drug-induced |
| 22 | 58 | F | 452.4 | 425.50 | Ischemic |
| 23 | 39 | M | 280.0 | 180.00 | Infectious |
| 24 | 44 | M | 185.0 | 165.00 | Drug-induced |
| 25 | 49 | M | 248.0 | 170.00 | Drug-induced (APAP) |
| 26 | 59 | M | 213.3 | 200.20 | Drug-induced |
| 27 | 28 | M | 660.6 | 774.00 | Infectious |
| 28 | 58 | M | 184.5 | 620.10 | Infectious |
| 29 | 50 | M | 203.7 | 220.61 | Infectious |
| 30 | 66 | F | 187.3 | 186.30 | Ischemic |
| 31 | 53 | M | 167.0 | 158.50 | Infectious |
| 32 | 22 | M | 177.0 | 162.20 | Drug-induced |
| 33 | 40 | M | 191.7 | 183.60 | Toxic |
| 34 | 36 | M | 235.9 | 269.80 | Infectious |
| 35 | 55 | M | 261.0 | 205.00 | Infectious |
| 36 | 48 | M | 365.8 | 216.60 | Infectious |

APAP, acetaminophen; ALT, alanine aminotransferase; AST, aspartate aminotransferase.

Table S2. The prediction details of each Lipin3-JAK1 protein complex.

| Protein-protein complex | △G (kcal.mol^-1^) | Kd (M) at 25℃ | ICs intermolecular | ICs charged-charged | ICs charged-polar | ICs charged-apolar | ICs polar-polar | ICs polar-apolar | ICs apolar-apolar | NIS charged | NIS apolar |
| --- | --- | --- | --- | --- | --- | --- | --- | --- | --- | --- | --- |
| Model 1 | -22.6 | 2.6e-17 | 288 | 34 | 37 | 70 | 17 | 49 | 81 | 31.84 | 37.14 |
| Model 2 | -18.6 | 2.4e-14 | 233 | 17 | 38 | 66 | 18 | 41 | 53 | 31.56 | 37.44 |
| Model 3 | -19.5 | 4.6e-15 | 240 | 26 | 38 | 69 | 11 | 34 | 62 | 31.71 | 37.14 |
| Model 4 | -7.5 | 3.1e-06 | 57 | 4 | 10 | 21 | 4 | 5 | 13 | 31.54 | 36.99 |
| Model 5 | -14.6 | 2.0e-11 | 129 | 30 | 16 | 37 | 1 | 15 | 30 | 32.5 | 35.43 |

△G, predicted binding affinity; Kd, predicted dissociation constant; ICs intermolecular, number of intermolecular contacts ; ICs charged-charged, number of charged-charged contacts; ICs charged-polar, number of charged-polar contacts; ICs charged-apolar, number of charged-apolar contacts; ICs polar-polar, number of polar-polar contacts; ICs polar-apolar, number of apolar-polar contacts; ICs apolar-apolar, number of apolar-apolar contacts; NIS charged, percentage of charged NIS residues; NIS apolar, percentage of apolar NIS residues.

Table S3. Primers used in this study

| Gene | Forward primer | Reverse primer | Species |
| --- | --- | --- | --- |
| *GSDME* | AGTCACTCTTCGTTTGGAACC | CTGAAGTACCAGGTTGTCCATATT | Mouse |
| *ACSL4* | ACCCCAGAGGTGAGATTGTG | AATATCGCCAGTGCAAAACC | Mouse |
